# Phosphorylated FAT10 Is More Efficiently Conjugated to Substrates, Does Not Bind to NUB1L, and Does Not Alter Degradation by the Proteasome

**DOI:** 10.3390/biomedicines12122795

**Published:** 2024-12-09

**Authors:** Jinjing Cao, Annette Aichem, Michael Basler, Gerardo Omar Alvarez Salinas, Gunter Schmidtke

**Affiliations:** 1 Division of Immunology, Department of Biology, University of Konstanz, 78457 Konstanz, Germany; jinjing.cao@uni-konstanz.de (J.C.); gerardo.alvarez@uni-konstanz.de (G.O.A.S.); 2 Institute of Cell Biology and Immunology Thurgau (BITg), University of Konstanz, 8280 Kreuzlingen, Switzerland; annette.aichem@bitg.ch (A.A.); michael.basler@uni-konstanz.de (M.B.)

**Keywords:** FAT10, phospho-mimetic FAT10, FAT10ylation, degradation, TRIM25, NUB1L, RPN10

## Abstract

**Background**: FAT10 is a member of the ubiquitin-like modifier family. Similar to ubiquitin, FAT10 has a distinct enzyme cascade consisting of E1-activating, E2-conjugating, and possibly several E3-ligating enzymes, which will covalently link FAT10 to substrate proteins in order to target them directly for proteasomal degradation. FAT10 was reported to be phosphorylated by IKKβ during infection with influenza A virus. **Methods**: To assess the difference between the FAT10-dependent degradation of phosphorylated FAT10 and the non-phosphorylated FAT10 wild type (FAT10 WT), a mutated FAT10 that mimicked phosphorylation (FAT10 D) was constructed by replacing several serine residues and one threonine residue with aspartic or glutamic acid. The FAT10 degradation or conjugation was compared between the phospho-mimetic FAT10 and the wild-type FAT10 with respect to the dependence of the E3 ligase TRIM25, the UBL-UBA protein NUB1L, and the proteasomal ubiquitin receptor RPN10. **Results**: The phospho-mimetic FAT10 was more efficiently conjugated to substrate proteins as compared to the wild-type FAT10, particularly if TRIM25 was co-expressed. Additionally, the phospho-mimetic FAT10 was not bound by NUB1L. However, this did not affect FAT10 D or FAT10 WT degradation. No differences were found in the binding affinity of phospho-mimetic FAT10 to RPN10. **Conclusions**: In brief, the phospho-mimetic FAT10 shows enhanced conjugation efficiency, but phosphorylation does not alter its degradation by the proteasome. This reveals that phosphorylation may fine-tune FAT10’s interactions with specific interaction partners without disrupting its core function of proteasomal degradation.

## 1. Introduction

Human leukocyte antigen (HLA)-F adjacent transcript 10 (FAT10) is a member of the ubiquitin-like modifier family, encoded in the major histocompatibility complex (MHC) class I region [1]. FAT10, a 165 aa multifunctional protein [2], consists of two tandemly arranged ubiquitin-like (UBL) domains (N- and C-domains) linked by a flexible linker (KPSDE) [3]. The N- and C-terminal UBL domains of FAT10 share 29% and 36% sequence identity with ubiquitin, respectively [4]. Similar to the ubiquitin cascade, FAT10 has a specific enzymatic cascade for directing its conjugates to proteasomal degradation [5,6]—a process termed “FAT10ylation” [7]. Several enzymes participate in the cascade, including the bispecific E1-activating enzyme UBA6 [8,9,10], the bispecific E2-conjugating enzyme USE1 [8,11,12], and putative E3 ligases such as Parkin [13,14,15]. So far, several proteins have been shown to interact with FAT10 through covalent or non-covalent interaction. Examples of covalently interacting proteins include p62, Parkin [13], and HUWE1 [16]. FAT10 plasticity destabilizes substrates significantly and creates partially unstructured regions in the substrate to enhance degradation [17]. Some reported non-covalently interacting proteins include histone deacetylase 6 (HDAC6), β-catenin [18,19], proliferating cell nuclear antigen (PCNA) [20], and Mfn2 [13]. These proteins play important roles in several biological processes related to FAT10 [21] and are possibly implicated in the role of FAT10 in disease development. Additionally, numerous studies have demonstrated a correlation between FAT10 and cancer [22,23,24,25,26,27,28,29,30,31,32].

FAT10 is predominantly expressed in the immune system, e.g., the thymus, lymph nodes, and spleen, whereas ubiquitin is expressed ubiquitously [33,34,35,36]. RIG-I [37] plays a key role in the innate immune response to RNA viruses. FAT10 inhibits RIG-I activation and impairs the type I interferon signaling cascade. Two E3 ligases—TRIM25 and ZNF598—are notable for their roles in the signaling pathway. TRIM25 stabilizes FAT10 bound to RIG-I and ZNF598 promotes the interaction between FAT10 and RIG-I [34,38]. Moreover, FAT10 is phosphorylated by IκB kinase β (IKKβ) in response to the pro-inflammatory cytokine tumor necrosis factor (TNF) and during influenza A virus infection at multiple serine (S) and threonine (T) residues (S62, S64, T77, S95, and S109) [39]. Additionally, the induction of type I IFN is decreased by phosphorylation of FAT10 [39]. Nevertheless, the effect of phosphorylation of FAT10 in the proteasomal degradation pathway remains unclear.

FAT10 targets substrates for degradation by the 26S proteasome, a multienzyme complex which comprises the 19S regulatory particle (RP, also called PA700) and a 20S cylindrical core particle (CP) [40,41]. NEDD8 ultimate buster-1 long (NUB1L) and RPN10 are key proteins in FAT10 degradation. NUB1L, an alternative splicing variant of NUB1, harbors three ubiquitin-associated (UBA) domains, as compared to the two UBA domains of NUB1, and one ubiquitin-like domain (UBL) [42,43]. NUB1L accelerates FAT10 degradation [44,45]. NUB1L utilizes FAT10’s intrinsic instability to trap its N-terminal ubiquitin-like domain in an unfolded state, facilitating degradation [46]. UBL-UBA domain proteins, such as RAD23, bind polyubiquitin and the proteasome and are an additional layer of regulated proteasomal degradation [47]. The UBA domains of NUB1L do not bind mono- or polyubiquitin but interact with the N-terminal UBL domain of FAT10, while the UBL domain of NUB1L binds to the von Willebrand A (VWA) domain of RPN10 [48]. RPN10 is a subunit of 19S RP and was the first reported polyubiquitin receptor [49,50,51]. FAT10 can on its own directly bind to the VWA domain of RPN10 to facilitate FAT10 degradation [52]. However, it is unknown how phosphorylated FAT10 interacts with NUB1L or RPN10 in the FAT10 degradation pathway.

Prompted by a previous study [39], we constructed a phospho-mimetic FAT10, which was used in this study. We aimed to elucidate the role of the phospho-mimetic FAT10 variant in the FAT10 degradation pathway, particularly with respect to its interaction with NUB1L.

## 2. Materials and Methods

### 2.1. Cell Culture and Cell Lines

Human HEK293T cells were cultured in DMEM (Gibco, Darmstadt, Germany) supplemented with 10% fetal bovine serum (Gibco, Darmstadt, Germany) and 1% penicillin–streptomycin (Gibco, Darmstadt, Germany). Tetracycline-regulated, NUB1L-overexpressing HEK293 cells were maintained under similar culture conditions [53,54]. All cells were sourced from our laboratory’s cell repository.

### 2.2. Plasmid Constructs

All clones were generated using the original plasmids listed in Tables S1 and S2. The original plasmids, such as pcDNA3.1-His-3xFLAG-FAT10 (FAT10 WT), pCMV-FLAG-TRIM25, and Human s5a, were obtained from our laboratory’s plasmid repository. Oligonucleotide primers were designed to be complementary to the opposite strands of the vector with the desired mutation. All primers are listed in Tables S1 and S2. For site-directed mutagenesis, the QuikChange Lightning Multi Site-Directed Mutagenesis Kit (Agilent, Heppenheim, Germany) and the Q5 Site-Directed Mutagenesis Kit (NEB, Frankfurt, Germany) manual. As for normal plasmid construction, the extension of primers by Phusion High-Fidelity DNA Polymerase (NEB, Frankfurt, Germany) generated a desired PCR product. Products and vectors were ligated using the Quick Ligation Kit (NEB, Frankfurt, Germany) following digestion with specific enzymes. The ligated constructs were transformed into competent E. coli, and isolated plasmids from bacterial colonies were sequenced.

### 2.3. Transient Transfection of Plasmids

Plasmids were transiently transfected into HEK293T cells using Polyethyleneimine (PEI; Polysciences, Hirschberg, Germany) at a 1:3 ratio of DNA to reagent dissolved in serum-free DMEM medium for 24 h.

### 2.4. Cycloheximide Chase (CHX) Experiments

CHX experiments were performed as described previously [3]. In brief, cells were treated with 50 µM cycloheximide and divided into aliquots, one of which was harvested immediately, while the remaining aliquots were harvested at indicated time points.

### 2.5. Immunoprecipitation and Immunoblotting

Immunoprecipitation and immunoblotting experiments were performed as previously described [3]. The antibodies used are listed in Table S3. In brief, cells were lysed and centrifuged, and the cleared supernatants were transferred into new reaction tubes. Then, 20 µL of protein G beads was added to each of the antibodies. After overnight incubation, the samples were centrifuged, the supernatants were separated, and the beads with bound proteins were washed five times and then subjected to boiling in SDS sample buffer. Next, SDS gel electrophoresis and immunoblotting followed by incubation with primary and secondary antibodies was performed.

### 2.6. Radiolabeling and Pulse–Chase Experiments

Radiolabeling and pulse–chase experiments were performed as previously described [54]. In brief, cells were starved for one hour in methionine- and cysteine-free medium. After starvation, the cells were pulsed for one hour with S35 Meth/Cys in medium. After the pulse, the radioactive medium was removed by washing the cells three times. The cells were resuspended in normal medium divided into aliquots and harvested at indicated time points. After lysis, immunoprecipitation and SDS gel electrophoresis gels were dried and exposed to phosphor imaging plates.

### 2.7. Statistical Analysis

All statistical analyses were performed using GraphPad Prism software (version 8.0). Error bars represent means ± SDs unless otherwise stated. The two-tailed unpaired Student’s t-test and one-way ANOVA with Tukey’s multiple-comparison test were used to compare data sets with two or more continuous variables, respectively.

## 3. Results

### 3.1. Phospho-Mimetic FAT10 Is More Efficiently Conjugated than Wild-Type FAT10

**Figure 1:**
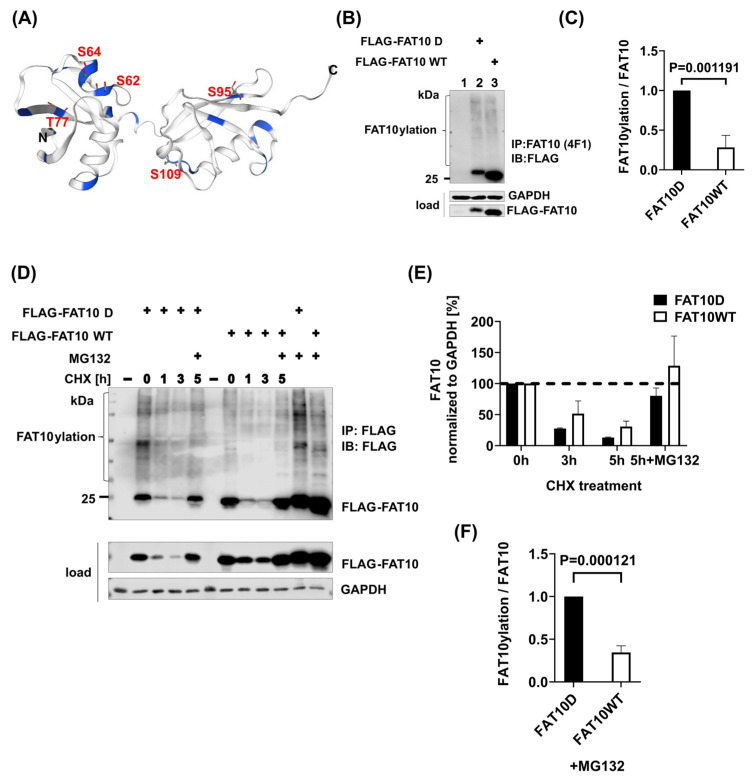
The phospho-mimetic FAT10 is more efficiently conjugated than wild-type FAT10. (**A**) FAT10 structure built in SWISS-MODEL. Blue represents all serine and threonine residues which might be phosphorylated. (**B**) HEK293T cells were transiently transfected with FLAG-FAT10 D or FLAG-FAT10 WT. One day later, cells were lysed, followed by FAT10 immunoprecipitation using a monoclonal FAT10-reactive antibody (4F1). SDS-PAGE and immunoblot analysis were performed with the indicated antibodies. (**C**) Quantification of the ECL signals from three independent experiments, as shown in (**B**). Values of FAT10ylation were normalized to the respective FLAG-FAT10 (WT or D) expression levels in the immunoprecipitates. The value of FLAG-FAT10 D was set to 1, and all other values were calculated accordingly. An unpaired Student’s t-test was used for statistical analysis. Error bars represent means ± SDs. (**D**) HEK293T cells were transiently transfected with constructs expressing the indicated forms of FLAG-FAT10, followed by a cycloheximide (CHX) chase for 5 h. Where indicated, cells were additionally treated for 5 h with MG132 (10 µM). The immunoprecipitation and immunoblot analyses were performed with the indicated antibodies. GAPDH was used as a loading control. (**E**) Quantification of the ECL signals of the lysates (loads) from three independent experiments, as shown in (**D**). Levels were normalized to the respective levels of the housekeeping gene GAPDH. Values at 0 h were set to unity, and the other values were calculated accordingly. (**F**) Quantification of the ECL signals obtained from cells only treated with MG132 in three independent experiments, as shown in (**D**). Values of FAT10ylation were normalized to the respective FLAG-FAT10 (WT or D) expression levels in the immunoprecipitates. The value of FLAG-FAT10 D was set to 1, and all other values were calculated accordingly. An unpaired Student’s t-test was used for statistical analysis. Error bars represent means ± SDs.

Phosphorylation of FAT10 at multiple sites (S62, S64, T77, S95, and S109) has been previously reported [39], and the positions of the sites are schematically shown in Figure 1A. Serine (S) and threonine (T) carry a negative charge after phosphorylation. To simulate such a charge, we generated a phospho-mimetic FAT10 mutant (FAT10 D), with S62, S64, S95, and S109 mutated to aspartic acid (D) and T77 mutated to glutamic acid (E). To investigate the impact of these modifications on FAT10’s role in protein degradation, we transiently transfected HEK293T cells with either wild-type FAT10 (FAT10 WT) or FAT10 D. The expression of FAT10, as well as its conjugate formation, was analyzed by immunoprecipitation and immunoblotting. The result indicated that FAT10 D, despite its lower expression rate, formed at least as many conjugates as FAT10 WT, yielding a higher conjugate-to-monomer ratio (Figure 1B,C). The lower expression rate of FAT10 D was not due to a faster degradation rate because FAT10 D was degraded at the same rate as FAT10 WT (Figure 1D,E). Notably, when cells were treated with the proteasome inhibitor MG-132, it was evident that FAT10 D exhibited a significantly higher conjugation efficiency than FAT10 WT (Figure 1D,F). Thus, it was suggested that phospho-mimetic FAT10 is more quickly or more efficiently conjugated to target proteins than wild-type FAT10.

### 3.2. TRIM25 Contributes to FAT10 Conjugation, Without Changing the Degradation

**Figure 2:**
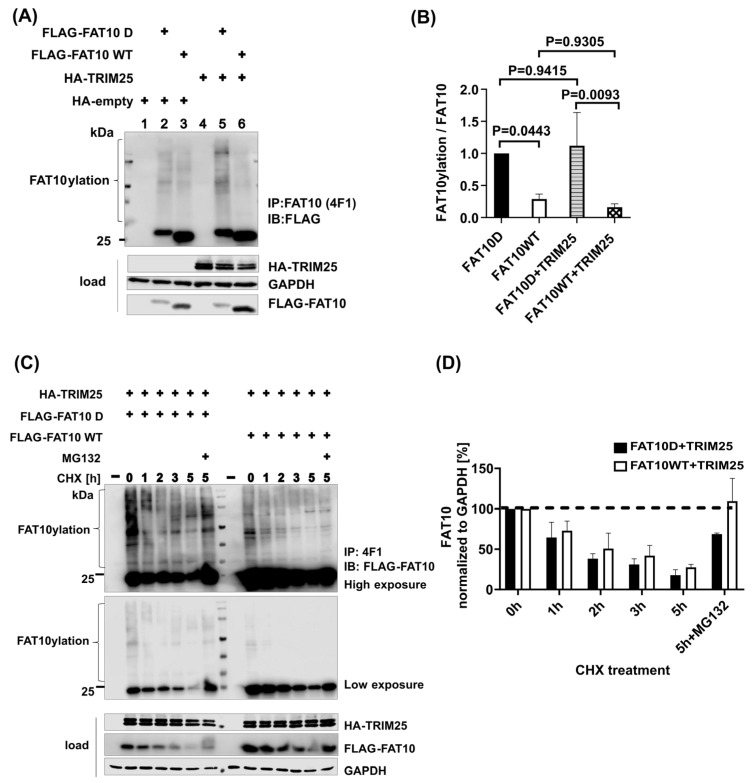
TRIM25 contributes to FAT10 expression but does not impact FAT10 degradation. (**A**) HEK293T cells were transiently co-transfected with TRIM25 and FAT10 expression plasmids. One day later, cells were lysed, followed by 4F1-immunoprecipitation, SDS-PAGE, and immunoblot analysis with the indicated antibodies. (**B**) Quantification of the ECL signals from three independent experiments, as shown in (**B**). Values of FAT10ylation were normalized to the respective FLAG-FAT10 (WT or D) expression levels in the immunoprecipitates. The value of FLAG-FAT10 D was set to 1, and all other values were calculated accordingly. A one-way ANOVA with Tukey’s multiple-comparison test was applied. Error bars represent means ± SDs. (**C**) HEK293T cells were transiently transfected with expression constructs for the indicated proteins, followed by a cycloheximide (CHX) chase for 5 h. Where indicated, cells were additionally treated for 5 h with MG132 (10 µM). Cells were lysed, followed by 4F1-immunoprecipitation, SDS-PAGE, and immunoblot analysis with the indicated antibodies. GAPDH was used as a loading control. (**D**) Quantification of the ECL signals of the lysates (loads) from three independent experiments, as shown in (**D**). Levels were normalized to the respective levels of the housekeeping gene GAPDH. Values at 0 h were set to unity, and the other values were calculated accordingly.

TRIM25, an E3 ubiquitin/ISG15 ligase, elicits RIG-I activation by inducing K63 polyubiquitination. A previous study demonstrated that FAT10 was recruited to RIG-I-TRIM25 to form an inhibitory complex, where FAT10 was stabilized by TRIM25 [38]. This prompted us to ask if there is any difference between FAT10 D and FAT10 WT concerning interaction with TRIM25. FAT10 and TRIM25 plasmids were co-transfected into HEK293T cells, and a cycloheximide chase was performed as described for Figure 1. FAT10 and potential conjugates were immune-precipitated and analyzed. As depicted in Figure 2, the co-expression led to a much better conjugation of FAT10 D as compared to FAT10 WT. There was no detectable influence of TRIM25 on the degradation rate of FAT10.

### 3.3. The Phospho-Mimetic FAT10 Alleviates the Binding of NUB1L

**Figure 3:**
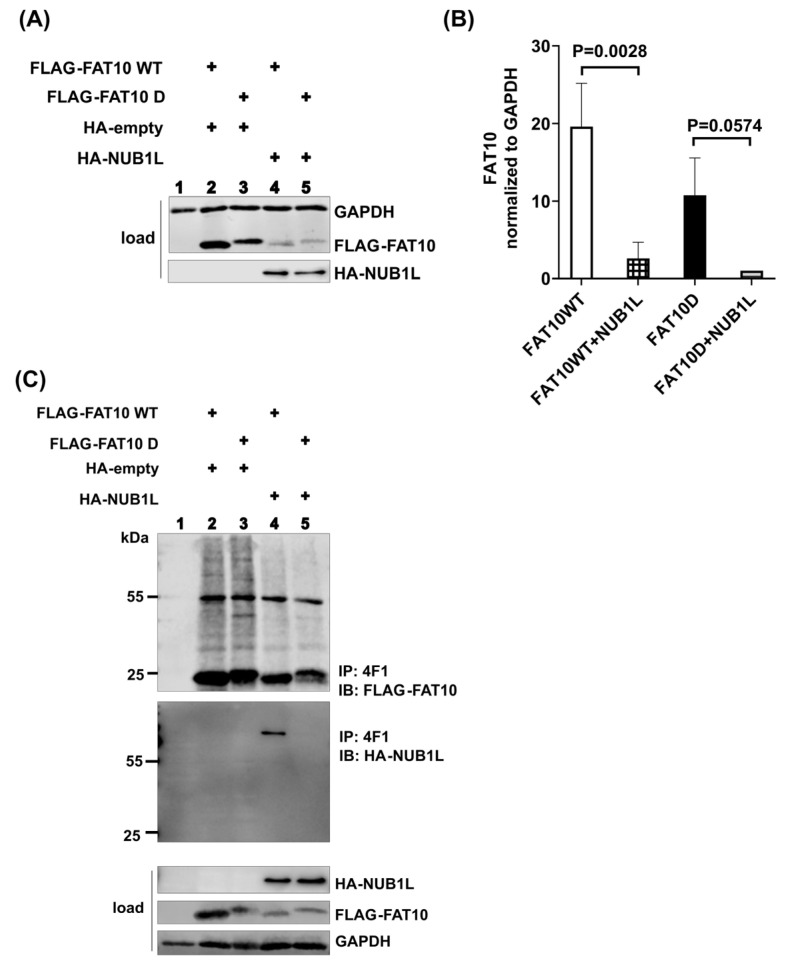
Phospho-mimetic FAT10 alleviates the binding of NUB1L. (**A**) HEK293T cells were transiently co-transfected with NUB1L and FAT10. One day later, cells were lysed, followed by SDS-PAGE and immunoblot analysis with the indicated antibodies. GAPDH was used as a loading control. (**B**) Quantification of the ECL signals of the lysates (loads) from three independent experiments, as shown in (**A**). Levels were normalized to the respective levels of the housekeeping gene GAPDH. The value of FLAG-FAT10 D was set to 1, and all other values were calculated accordingly. Statistical analysis was performed by one-way ANOVA with Tukey’s multiple-comparison test. Error bars represent means ± SDs. (**C**) HEK293T cells were transiently co-transfected with NUB1L and FAT10 expression plasmids. One day later, cells were lysed, followed by immunoprecipitation (4F1 + protein A beads), SDS-PAGE, and immunoblot analysis with the indicated antibodies. GAPDH was used as a loading control.

**Figure 4:**
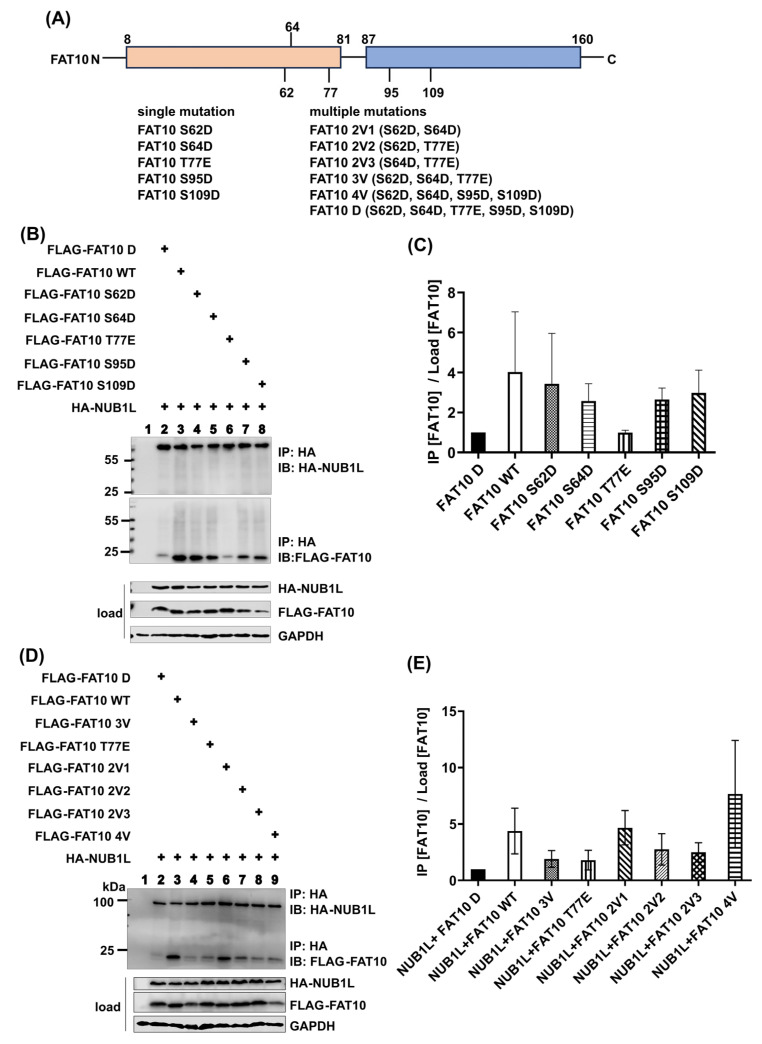
FAT10 threonine 77 is important in mediating FAT10–NUB1L interactions. (**A**) Scheme showing the protein domain of FAT10 and different mutations. (**B**,**D**) HEK293T cells were transiently co-transfected with NUB1L and FAT10. One day later, cells were lysed, followed by HA-immunoprecipitation, SDS-PAGE, and immunoblot analysis performed with the indicated antibodies. GAPDH was used as a loading control. (**C**,**E**) Quantification of the ECL signals from three independent experiments, as shown in (**B**) or (**D**). The ratio values represent FLAG-FAT10 expression levels in the immunoprecipitates relative to the respective FLAG-FAT10 expression levels in the lysates (loads). The value of FLAG-FAT10 D was set to 1, and all other values were calculated accordingly.

**Figure 5:**
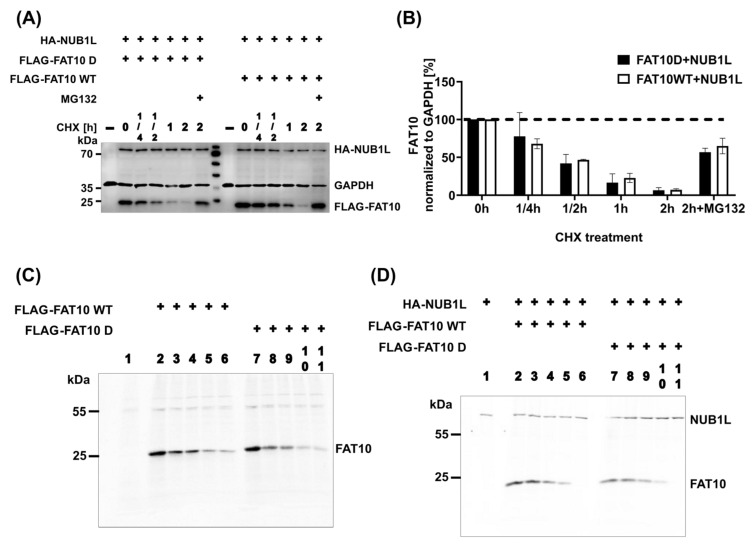
FAT10 D exhibits comparable degradation kinetics to FAT10 WT. (**A**) HEK293T cells were transiently transfected with constructs expressing the indicated plasmids, followed by a cycloheximide (CHX) chase for 5 h. Where indicated, cells were additionally treated for 5 h with MG132 (10 µM). Cells were lysed, followed by SDS-PAGE and immunoblot analysis with the indicated antibodies. GAPDH was used as a loading control. (**B**) Quantification of the ECL signals of the lysates (loads) from three independent experiments, as shown in (**A**). Levels were normalized to the respective levels of the housekeeping gene GAPDH. Values at 0 h were set to unity, and the other values were calculated accordingly. A stable cell line (overexpressing NUB1L) was transiently transfected with FAT10 D or FAT10 WT and treated with (**D**) or without (**C**) tetracycline. Following this, cells were subjected to a radioactive pulse–chase experiment.

FAT10 was reported to interact with the UBA domains of NUB1L [44]. To investigate whether FAT10 phosphorylation affects this interaction, we co-transfected HEK293T cells with either FAT10 WT or FAT10 D and NUB1L. Co-expression of NUB1L with FAT10 D or FAT10 WT resulted in a significant reduction in detectable FAT10 (Figure 3A,B). It was reported that NUB1L can bind to FAT10 and accelerate its degradation [54]. This observation is compatible with NUB1L-mediated accelerated FAT10 degradation. To investigate this in more detail, we first intended to detect binding of FAT10 D or FAT10 WT to NUB1L. Only in the case of FAT10 WT did co-expression and IP result in a co-precipitated band of NUB1L (Figure 3C). Apparently, FAT10 D lost its ability to interact with NUB1L. There are reports that phosphorylation of ubiquitin and NEDD8 at a single residue (S65 in both cases) [55,56] changed their structure and function. We reported evidence of the phosphorylation of FAT10 at different sites [39], among them serine 64. To investigate whether a single phosphorylation site in FAT10 is sufficient to diminish the FAT10–NUB1L interaction, single and multiple mutants were generated and analyzed. As can be seen in Figure 4, the mutation of threonine 77 to glutamic acid had the most significant effect and was sufficient to almost prevent the binding of FAT10 to NUB1L. We reported previously that the interaction between NUB1L and FAT10 is not required for the accelerated degradation of FAT10 by NUB1L [45]. We analyzed the degradation rates of FAT10 WT and D in the presence of NUB1L in a cycloheximide chase experiment. As can be seen in Figure 5A,B, there was no significant difference in the rates of degradation of FAT10 WT and FAT10 D in the presence of NUB1L. To exclude any influence that the translation blocker cycloheximide might have on cellular processes, we performed a site-by-site radioactive pulse–chase experiment on FAT10 WT and FAT10 D with and without NUB1L. There was no obvious difference detectable in the rates of degradation of FAT10 WT and FAT10 D in the presence or absence of NUB1L (Figure 5C,D). However, the degradation of both FAT10 D and FAT10 WT was about 2-fold faster in the presence of NUB1L. This confirms our previous report about the independence of accelerated degradation and binding of FAT10 and NUB1L.

### 3.4. The Phospho-Mimetic FAT10 Does Not Change the Binding of RPN10

**Figure 6:**
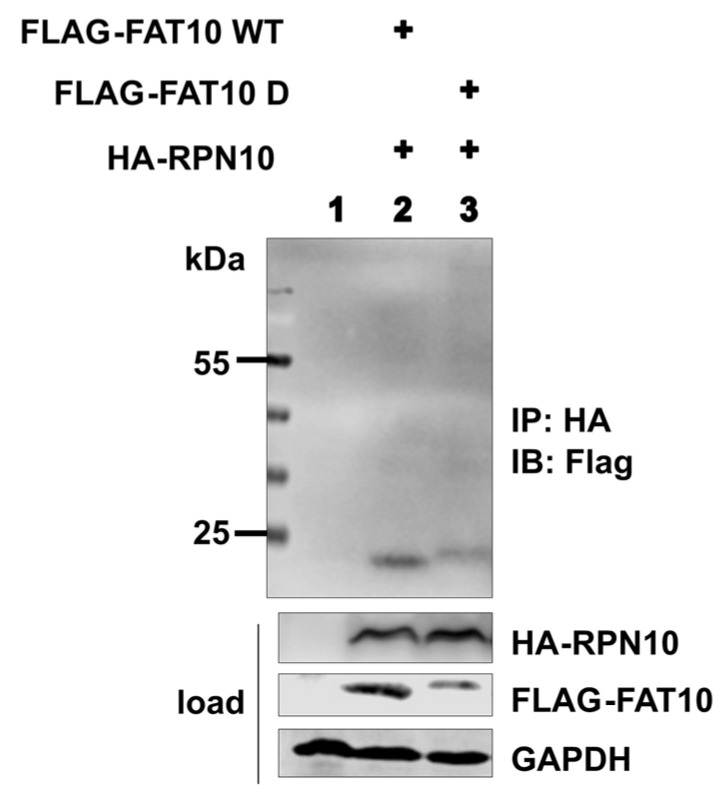
The phospho-mimetic FAT10 D does not change the binding of RPN10. HEK293T cells were transiently co-transfected with RPN10 and FAT10. One day later, cells were lysed, followed by immunoprecipitation (HA beads), SDS-PAGE, and immunoblot analysis with the indicated antibodies. GAPDH was used as a loading control.

FAT10 not only binds with its N-terminal UBL domain to the UBA domains of NUB1L, but it also interacts with the VWA domain of RPN10 via its C-terminal UBL domain for protein degradation [52]. We examined whether the phospho-mimetic mutations altered FAT10’s interaction with RPN10. Co-transfection of HEK293T cells with FAT10 WT or FAT10 D and RPN10, followed by immunoprecipitation, showed no significant difference in the interaction between FAT10 WT or FAT10 D and RPN10 (Figure 6). This indicates that despite the alleviated interaction with NUB1L, FAT10 D can still bind to RPN10 to mediate protein degradation, suggesting that phosphorylation of FAT10 does not impair its degradation function.

## 4. Discussion

Previous reports have shown that not only ubiquitin [55,57], SUMO [58], and NEDD8 [56] can be phosphorylated, but also FAT10 can be phosphorylated by IKKβ upon TNF stimulation and during influenza A virus infection [39]. Phosphorylation of FAT10 has been shown to limit IFN-I production during inflammation. Phosphorylation of ubiquitin and NEDD8 occurs on serine 65 [55,56], enabling interactions with specific binding proteins. Phospho-ubiquitin activates the E3 ligase Parkin, leading to faster conjugation of ubiquitin from the loaded E2 UBCH7, while phospho-NEDD8 leads to activation of HSP70 [55,56]. We constructed a phospho-mimetic FAT10 (FAT10 D, S62D, S64D, T77E, S95D, and S109D) by substituting serine and threonine residues with aspartic acid and glutamic acid, respectively (Figure 1A). We chose this approach because it was also used in the phospho-ubiquitin–parkin study [55]. FAT10 D, despite its lower expression, yielded at least the same amount of conjugated FAT10 as FAT10 WT, leading to a much higher conjugate-to-monomer ratio (Figure 1B,C). The rate of degradation was not different between FAT10 D and FAT10 WT (Figure 1D,E).

In the type I interferon signaling cascade, TRIM25 is beneficial for stabilizing FAT10 [38]. Phosphorylated FAT10 has been shown to reduce TRAF3 ubiquitination, hindering interferon regulatory factor 3 (IRF3) phosphorylation and downstream IFN-I induction [39]. To assess the interaction with TRIM25 after FAT10 is phosphorylated, FAT10 D and WT and TRIM25 were co-transfected into HEK293T cells. As can be seen in Figure 2A,B, the difference in the conjugate formation of FAT10 D and FAT10 WT was even more pronounced in the presence of TRM25. This seems to be analogous to phospho-ubiquitin, which also activates an E3, parkin [55]. The rate of degradation was not changed in the presence of TRIM25 (Figure 2C,D). Additionally, the E1 and E2 enzymes of FAT10 were transfected with FAT10 and TRIM25 into UBA6 KO and USE1 KO cells. The results revealed that UBA6 and USE1 are indispensable for FAT10ylation (Figure S1).

Studies have shown that FAT10 targets substrates for degradation by the 26S proteasome and that NUB1L accelerates the process [40,41,42,43,44,45]. NUB1L can bind FAT10 via its UBA domains; however, for accelerated FAT10 degradation, the UBL domain of NUB1L is sufficient and the binding of FAT10 is dispensable [45]. In the current study, HEK293T cells were co-transfected with FAT10 and NUB1L. Transfection with NUB1L resulted in a reduction in detectable FAT10, independently whether FAT10 WT or FAT10 D was analysed. (Figure 3A,B). We tested whether FAT10 D could still be co-immunoprecipitated with NUB1L but failed to detect an interaction between FAT10 D and NUB1L, unlike FAT10 WT, which bound NUB1L (Figure 3C). To exclude the possibility that FAT10 D was somehow differently folded, we used FAT10 variants with only a single amino acid changed to mimic phosphorylation, as well as some combinations (Figure 4A). The experiments identified Thr77 as a key residue for this interaction (Figure 4B–E). As soon as Thr 77 carried a negative charge, the interaction with NUB1L was strongly diminished. This is analogous to ubiquitin and NEDD8, where a single-site phosphorylation is sufficient to change an interaction [56]. NUB1L was reported to accelerate the degradation of FAT10 [54], and this was independent of FAT10 binding to NUB1L [45]. The analysis of the half-lives of FAT10 D and FAT10 WT in cycloheximide chase experiments did not reveal any differences. To exclude any effect of the translational inhibitor cycloheximide, we used a radioactive pulse–chase labeling approach with and without NUB1L. As shown in Figure 5C,D, FAT10 and FAT10 D behaved indistinguishably. Their half-lives were the same, and the acceleration by NUB1L was the same as well. This confirms the data reported by Schmidtke [45] on the independence of the accelerated degradation from the binding. Furthermore, the results are in agreement with reports about other UBL-UBA domain proteins, such as Rad23 and Dsk2 [59,60]. These proteins bind polyubiquitinated proteins and act as soluble polyubiquitin carriers to the 26S proteasome, defining a new layer of substrate specificity [47]. Reported functions of the UBL-UBA proteins include inhibition of excess polyubiquitination, prevention of deubiquitination, activation of the 26 S complex, and positioning of the substrate in a favorable position for degradation. The fact that NUB1L prefers the binding of unphosphorylated FAT10 may point towards a new level of regulation. NUB1L leads to faster degradation of FAT10 and its conjugates. Overexpression of FAT10 is toxic in some cell lines [61], so NUB1L may reduce the toxicity of FAT10 by removing it. However, conjugation of FAT10 is also required. The phosphorylation of FAT10 may prevent it from binding to NUB1L, leaving it available for conjugation. After conjugation, the substrate is still removed more quickly in the presence of NUB1L. The UBL domain of NUB1L interacts with the von Willebrand A (VWA) domain of the proteasomal subunit RPN10 for FAT10 degradation [48]. FAT10 itself can directly interact with the VWA domain of RPN10 to enable its degradation [52]. The interaction between FAT10 and RPN10 was not affected by the phospho-mimetic mutations (Figure 6), suggesting that FAT10’s degradation pathway remains intact even with altered NUB1L binding, explaining why there was no difference in the degradation of FAT10 D as compared to FAT10 WT. This was also proof that FAT10 D was correctly folded, as the binding to RPN10 and degradation by the proteasome were indistinguishable from those of FAT10 WT. There have been at least three reports in recent years providing evidence that FAT10 induces cancer by the stabilization of different proteins [29,62,63]. The mechanism is possibly the prevention of ubiquitylation by blocking lysine residues in the respective proteins. Apparently, degradation via the ubiquitin pathway is faster with respect to the FAT10 pathway. That is why an upregulated conjugation with FAT10 without faster degradation may be important in cancer development. More FAT10ylated protein means fewer substrates available for ubiquitylation and therefore a stabilization of certain proteins, which may contribute to cancer development.

## 5. Conclusions

In conclusion, while phospho-mimetic FAT10 exhibits increased conjugation efficiency, it does not alter the overall degradation process of itself or its conjugates. This suggests that phosphorylation may fine-tune FAT10’s interactions with specific partners without disrupting its core function in proteasomal degradation.

## Data Availability

All necessary data generated or analyzed during this study are presented in this article, and additional data can be made available by the corresponding author upon request.

## Supplementary Materials

The following supporting information can be downloaded at: https://www.mdpi.com/article/10.3390/biomedicines12122795/s1, Table S1: Site-directed mutagenesis, Table S2: Normal construction, Table S3: Reagents, Figure S1: UBA6 and USE1 are indispensable for FAT10ylation.
