# Supplementary material for: Phosphorylated FAT10 Is More Efficiently Conjugated to Substrates, Does Not Bind to NUB1L, and Does Not Alter Degradation by the Proteasome
Source: Biomedicines. 2024 Dec 9;12(12):2795. doi: 10.3390/biomedicines12122795 (PMC11673000; doi:10.3390/biomedicines12122795)
Supplement: Supplementary file 1 [file biomedicines-12-02795-s001.zip › biomedicines-3357148-supplementary.pdf]

Supplementary Materials

Table S1. Site-directed mutagenesis

Constructed plasmids	Primers	Templates
**FAT10 D** (S62D, S64D, T77E, S95D, S109D)	F1: 5′-GATCTTAAAGCCACGGAGAGACCTCGATTCTTATGGCATTGACAAAG-3′<br>F2: 5′-GAAGACCATCCACCTTGAGCTGAAAGTGGTGAAGCCCAG-3′<br>F3: 5′-CCCTTGTTTCTTGTGGAGGATGGTGATGAGGCAAAGAGGC-3′<br>F4: 5′-CTCCAGGTGCGAAGGGACAGCTCAGTGGCACAAG-3′	pcDNA3.1-His-3xFLAG-FAT10 (FAT10 WT)
**FAT10 S62D**	F: 5′- GCC ACG GAG AGA CCT CTC ATC TTA TG-3′<br>R: 5′- TTT AAG ATC TTG GAG CCC-3′	
**FAT10 S64D**	F: 5′- GAG AAG CCT CGA TTC TTA TGG CAT TGA C-3′<br>R: 5′- CGT GGC TTT AAG ATC TTG-3′	
**FAT10 T77E**	F: 5′- GAAGACCATCCACCTTGAGCTGAAAGTGGTGAAGCCCAG -3′<br>R: 5′- TCT TTG TCA ATG CCA TAA GAT GAG-3′	
**FAT10 S95D**	F: 5′- CCCTTGTTTCTTGTGGAGGATGGTGATGAGGCAAAGAGGC -3′<br>R: 5′- CAG CTC CTC ATC ACT GGG CT-3′	
**FAT10 S109D**	F: 5′- CTCCAGGTGCGAAGGGACAGCTCAGTGGCACAAG -3′<br>R: 5′- GAG GTG CCT CTT TGC CTC ATC -3′	
**FAT10 2V1** (S62, 64D)	F: 5′-GATCTTAAAGCCACGGAGAGACCTCGATTCTTATGGCATTGACAAAG -3′<br>R: 5′- TTGGAGCCCAGCAAAAGA-3′	
**FAT10 2V2** (S62D, T77E)	F: 5′- GAAGACCATCCACCTTGAGCTGAAAGTGGTGAAGCCCAG -3′<br>R: 5′- TCT TTG TCA ATG CCA TAA GAT GAG-3′	**FAT10 S62D**
**FAT10 2V3** (S64D, T77E)	F: 5′- GAAGACCATCCACCTTGAGCTGAAAGTGGTGAAGCCCAG -3′<br>R: 5′- TCT TTG TCA ATG CCA TAA GAT GAG-3′	**FAT10 S64D**
**FAT10 3V** (S62D, S64D, T77E)	F: 5′- GAAGACCATCCACCTTGAGCTGAAAGTGGTGAAGCCCAG -3′<br>R: 5′- TCT TTG TCA ATG CCA TAA GAT GAG-3′	**FAT10 2V1** (S62, 64D)
**FAT10 4V** (S62D, S64D, S95D, S109D)	F: 5′- CATCCACCTTACCCTGAAAGTGGTGA-3′<br>R: 5′- GTCTTCTCTTTGTCAATGC-3′	**FAT10 D** (S62D, S64D, T77E, S95D, S109D)

Table S2. Normal construction

Constructed plasmid	Primers	Original plasmid and restriction sites
HA-TRIM25	F: 5′-GAAACGGTACCTATGGCAGAGCTGTGC-3′<br>R: 5′-GTTTCTCTAGACCTACTTGGGGGAGCA-3′	pCMV-FLAG-TRIM25 and pcDNA-HA-hFAT10<br>Kpn I and Xba I
HA-RPN10	F: 5'-GAA ACG GTA CCT ATG GTG TTG GAA-3'<br>R: 5'-GTT TCT CTA GAC TCA CTT CTT GTC-3'	Human s5a and pcDNA-HA-hFAT10<br>Kpn I and Xba I

Table S3. Reagents

Reagents or kit	Company	Cat No.
EZview™ Red Protein A Affinity Gel	Millipore	P6486
EZview™ Red Anti-HA Affinity Gel	Millipore	E6779
EZview™ Red ANTI-FLAG® M2 Affinity Gel	Millipore	F2426
Monoclonal ANTI-FLAG® M2 antibody produced in mouse	Sigma-Aldrich	F1804
Monoclonal ANTI-FLAG® M2-Peroxidase (HRP) antibody produced in mouse	Sigma-Aldrich	A8592
Anti-GAPDH antibody produced in rabbit	Sigma-Aldrich	G9545
Anti-HA−Peroxidase antibody, Mouse monoclonal	Sigma-Aldrich	H6533
Monoclonal ANTI- FAT10 antibody produced in mouse (4F1)	Our laboratory	-
IRDye® 800CW Goat anti-Rabbit IgG Secondary Antibody	LICOER	926-32211
IRDye® 800CW Goat anti-Mouse IgG Secondary Antibody	LICOER	926-32210
IRDye® 680RD Goat anti-Rabbit IgG Secondary Antibody	LICOER	926-68071
IRDye® 680RD Goat anti-Mouse IgG Secondary Antibody	LICOER	926-68070
cOmplete™, EDTA-free Protease Inhibitor Cocktail	Roche	4693132001
β-mercaptoethanol	ROTH	4227.3
DMEM, high glucose, GlutaMAX™ Supplement	Gibco	61965026
Fetal Bovine Serum	Gibco	10270106
Penicillin-Streptomycin	Gibco	15140122
QuikChange Lightning Multi Site-Directed Mutagenesis Kit	Agilent	210513
Q5® Site-Directed Mutagenesis Kit	NEB	E0552S
Phusion® High-Fidelity DNA Polymerase	NEB	M0530S
Quick Ligation™ Kit	NEB	M2200S
Intercept® (TBS) Blocking Buffer	LICOER	927-60001
MG-132	Sigma-Aldrich	474787
Cycloheximide (CHX)	Sigma-Aldrich	239763
SuperSignal™ Western Blot Substrate Bundle, Pico PLUS	Thermo Scientific	A43840

All applications are based on the manufacturing processes of the company.

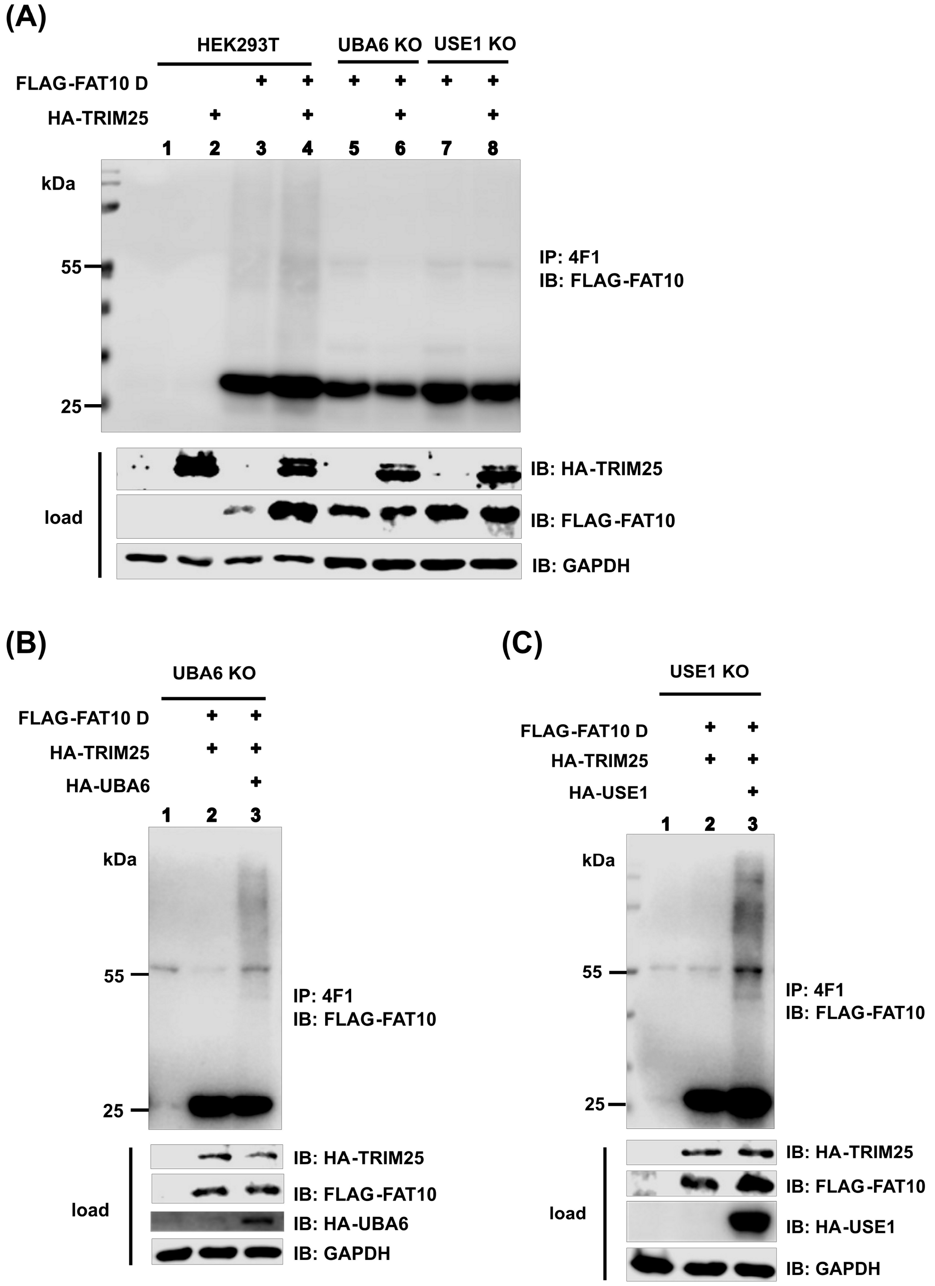

Figure S1. UBA6 and USE1 are indispensable for FAT10ylation. (A) HEK293T, UBA6 KO and USE1 KO cells were transiently transfected with TRIM25 and/or FAT10 D expression plasmids. One day later, cells were lysed, followed by 4F1-immunoprecipitation, SDS-PAGE, and immunoblot analysis with the indicated antibodies. GAPDH was used as a loading control. (B) UBA6 KO cells were transiently transfected with FAT10 D, TRIM25 and/or UBA6 expression plasmids. One day later, cells were lysed, followed by 4F1-immunoprecipitation, SDS-PAGE, and immunoblot analysis with the indicated antibodies. GAPDH was used as a loading control. (C) USE1 KO cells were transiently transfected with FAT10 D, TRIM25 and/or USE1 expression plasmids. One day later, cells were lysed, followed by 4F1-immunoprecipitation, SDS-PAGE, and immunoblot analysis with the indicated antibodies. GAPDH was used as a loading control.
